# Use of Cannabidiol in the Treatment of Epilepsy: Efficacy and Security in Clinical Trials

**DOI:** 10.3390/molecules24081459

**Published:** 2019-04-12

**Authors:** Serena Silvestro, Santa Mammana, Eugenio Cavalli, Placido Bramanti, Emanuela Mazzon

**Affiliations:** IRCCS Centro Neurolesi “Bonino-Pulejo”, Via Provinciale Palermo, Contrada Casazza, 98124 Messina, Italy; silvestro9110@gmail.com (S.S.); santa.mammana@irccsme.it (S.M.); eugenio.cavalli@irccsme.it (E.C.); placido.bramanti@irccsme.it (P.B.)

**Keywords:** cannabidiol, treatment-resistant epilepsy, clinical trials

## Abstract

Cannabidiol (CBD) is one of the cannabinoids with non-psychotropic action, extracted from *Cannabis sativa*. CBD is a terpenophenol and it has received a great scientific interest thanks to its medical applications. This compound showed efficacy as anti-seizure, antipsychotic, neuroprotective, antidepressant and anxiolytic. The neuroprotective activity appears linked to its excellent anti-inflammatory and antioxidant properties. The purpose of this paper is to evaluate the use of CBD, in addition to common anti-epileptic drugs, in the severe treatment-resistant epilepsy through an overview of recent literature and clinical trials aimed to study the effects of the CBD treatment in different forms of epilepsy. The results of scientific studies obtained so far the use of CBD in clinical applications could represent hope for patients who are resistant to all conventional anti-epileptic drugs.

## 1. Introduction

**Figure 1:**
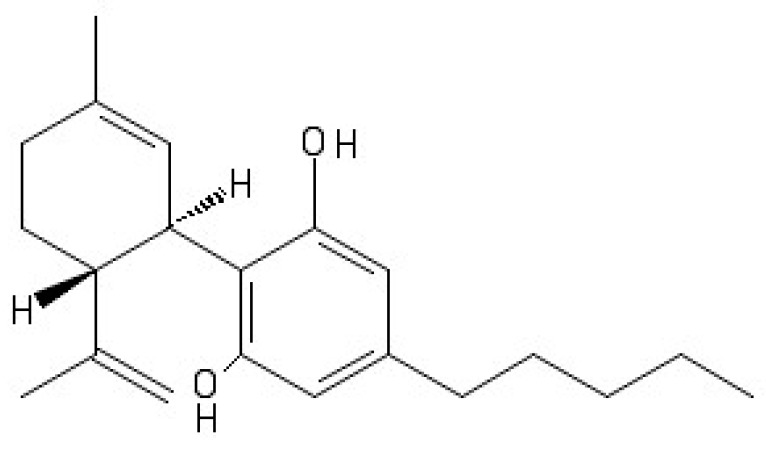
Structure of CBD.

*Cannabis sativa* L. is an ancient medicinal plant wherefrom over 100 cannabinoids are extracted [1]. Among them, the most studied are Δ^9^–tetrahydrocannabinol (Δ^9^–THC), a psychoactive compound, and the CBD, a non-psychotropic phytocannabinoid [2]. CBD is a cyclohexene which is substituted in position 1 by a methyl group, by a 2,6-dihydroxy-4-pentylphenyl group at position 3, and with a prop-1-en-2-yl group at position 4 (Figure 1). Most cannabinoids exert their action by interacting with cannabinoid receptors, but CBD shows a low affinity for these receptors. Nevertheless, it affects the activity of other receptors such as serotonin receptors [5-HT], opioid receptors [ORs], and non-endocannabinoid G protein-coupled receptors (GPCRs) [3] and other targets (ion channels and enzymes).

In recent years, the scientific community has shown interest in this compound due to its good safety profile and neuroprotective properties [4] in several neurodegenerative diseases, including Amyotrophic Lateral Sclerosis [5], Parkinson’s [6,7], Huntington’s [8] and Alzheimer’s diseases [9,10,11]. This neuroprotective action is due to its anti-inflammatory [12,13] and antioxidant [14,15] properties. CBD shows anti-inflammatory properties in several experimental studies, modulating some pro-inflammatory cytokines such as interleukin-1β (IL-1β ) [16], interleukin-6 (IL-6) [17,18] and tumor necrosis factor α (TNF-α) [16,18], as well as regulation of cell cycle and immune cells’ functions [19]. Furthermore, another mechanism by which CBD performs its anti-inflammatory action is mediated by interaction with the Transient Potential Vanilloid Receptor Type 1 (TRPV1). TRPV1 receptor is a nonselective cation channel that, when activated, allows the influx of Ca^2+^. The sensitivity but also the density of TRPV1 is increased during neuro-inflammatory conditions. The binding of CBD to TRPV1 leads to a desensitization of these receptors, with a consequent reduction in inflammation [20]. The CBD also carry out a potent antioxidant activity, modulating the expression of inducible nitric oxide synthase and nitrotyrosine as well as reducing production of reactive oxygen species [21]. CBD is also generating interest due to its therapeutic properties such as antidepressant [22], antipsychotic [23], analgesic [24], and antitumor [25]. In addition, it has been shown that CBD can significantly reduce two important forms of anxiety, namely obsessive-compulsive disorder [26] and post-traumatic stress disorder [27,28].

Moreover, for a long time, the CBD has been investigated for its anticonvulsant effects [29,30,31]. Several studies confirmed its efficacy in the treatment of epileptic seizures, especially in pediatric age [32,33]. In 2016, the first results of phase III clinical trials showed beneficial effects of CBD (Epidiolex^®^; GW Pharmaceutical, Cambridge, UK) in treatment-resistant seizure disorders, including Lennox-Gastaut Syndrome (LGS) and Dravet syndromes (DS).

Epilepsy is a chronic neurological disorder. About 30% of epilepsy patients are affected by Treatment-Resistant Epilepsy (TRE) due to the failure of common anti-epileptic therapies [34]. This form of epilepsy is characterized by recurrent seizures that negatively affect the quality of life. 

The purpose of this review is to provide an overview of recent clinical trials registered on ClinicalTrial.gov. These trials study the use of different CBD formulation in patients affected by severe forms of drug-resistant epilepsy. Moreover, we have described studies approved by local ethics committees published in PubMed.

## 2. Epilepsy

According to the World Health Organization, epilepsy affects more than 50 million people worldwide. Epilepsy is the most common neurological disorders characterized by recurrent seizures [35]. A “seizure” is a paroxysmal transient phenomenon determined by an abnormal excessive or synchronous neuronal activity in the brain [36]. Epilepsy can also cause deficit sensorimotor, cognitive, compromising quality of life and an increased risk of premature death [37]. The International League Against Epilepsy, according to the point of onset, classifies epileptic seizures into focal, generalized and unknown seizures [38]. Focal convulsions caused by an anomalous electrical activity in a circumscribed part of the brain and are classified into simple and complex. Simple focal convulsions are characterized by motor, sensory and sensory manifestations without loss of consciousness. On the contrary, complex focal convulsions involve a loss of consciousness [39]. Generalized seizures begin in one or more areas of the brain and can then spread to the entire brain. Generalized seizures are divided into crises absences, characterized by a rapid and transient loss of consciousness; tonic crises that cause muscle stiffening; atonic crisis, characterized by loss of muscular control; clonic seizures that cause rhythmic muscle movements; myoclonic seizures, characterized by muscle contraction and localized tremors. Finally, tonic-clonic seizures represent the most serious type of epileptic seizures, last about 5–10 min and are characterized by intense generalized contractions to the whole body [39,40]. The unknown seizures are called so when the beginning of a seizure is not known. These seizures can also be defined as "epileptic spasms" characterized by sudden extension or flexion of the limbs. Is defined Secondary Epilepsy when the onset is caused by several factors such as head trauma, infectious diseases (meningitis, AIDS, viral encephalitis), developmental disorders, alcohol or drug abuse, and other pathological conditions (brain tumors, stroke). 

The most well-known epilepsies are DS, Sturge-Weber Syndrome (SWS), Tuberous Sclerosis Complex (TSC) and West Syndrome (WS) and LGS. DS is a rare encephalopathy, which has its onset in the first year of life [41]. DS is associated with the mutation in the gene encoding the α1 subunit of the voltage gated sodium channel (*SCN1A*) [42]. SWS is caused by a somatic mutation of the *GNAQ* gene (9q21) that encodes the G_q_ protein, involved in the intracellular signal of several G protein-coupled receptors that control the function of various growth factors and vasoactive peptides [43]. Patients manifest neurological abnormalities of variousdegrees, focal epileptic seizures [44]. TSC is an autosomal dominant disease, caused by a mutation of two genes: *TSC1* (localized on chromosome 9p34.3) that encodes for hamartin and *TSC2* (localized on chromosome 16p13.3) that encodes for tubulin. Often TSC patients present generalized epilepsy. WS or Infantile Spasm (IS) is the epileptic encephalopathy. This syndrome is characterized by genetic heterogeneity and the mutated gene most frequently observed in patients with this syndrome is *CDKL5* (cyclin-dependent kinase-like 5) [45]. WS is characterized by the association between axial spasm discharges and psychomotor retardation [46]. LGS is a severe epileptic encephalopathy of childhood. This syndrome is a rare condition likely associated with a genes mutation. Nevertheless, to date, it is quite unclear how the involved genes may cause this syndrome mainly characterized by recurrent seizures from early in life. An epileptic form that does not respond to therapy with at least two or three appropriately selected anti-epileptic drugs (AEDs) is defined as TRE and this is estimated to affect 30% of patients [47,48].

## 3. Common Antiepileptic Drugs

AEDs are the mainstay for the treatment of epilepsy and are intended to mitigate seizures. Epileptogenic discharges occur as a result of neuronal hyperexcitability caused by voltage-dependent ion channels and neurotransmitter concentrations alteration. AEDs primarily act reducing neuronal excitability blocking excitatory neurotransmitter action such as glutamic acid and enhancing inhibitory neurotransmitters such as γ-aminobutyric acid (GABA). Furthermore, the antiepileptic actions of most AEDs are due to the modulation of voltage-gated ion channels such as sodium (Na^+^) and calcium (Ca^2+^). The neuronal Na^+^ and Ca^2+^ channels are responsible for the rise of the action potential and for the intrinsic excitability control of the neuronal system [49]. Some AEDs act inactivating a single voltage-dependent channel while others instead simultaneously inactivate bothchannels. Both of these mechanisms result in a reduction for neuronal hyperexcitability. Examples of drugs that perform interacting with a single channel are phenytoin that selectively blocks the Na^+^ channel [50] and ethosuximide that blocks the T-type Ca^2+^ channel [51]. Instead, carbamazepine, lamotrigine, oxcarbazepine and zonisamide control seizures blocking both these voltage-dependent ion channels [52].

There are also anti-epileptics that act enhancing the GABAergic system. GABA is the main inhibitory neurotransmitter of the nervous system that acts on GABA receptors, ligand-dependent ionic receptors that increase chlorine conductance. AEDs are responsible for increasing GABA transmission reducing neuronal excitability. Drugs that exert their action through these mechanisms are benzodiazepines, phenobarbital, stiripentol, tiagabine and vigabatrin. Benzodiazepines (such as clobazam diazepam, lorazepam, clonazepam) phenobarbital and stiripentol enhance the inhibitory transmission of GABA by allosteric activation of the GABA_A_ receptor thus increasing the frequency of chloride (Cl^−^) channel openings [53,54]. Vigabatrin, instead, is an inhibitor of GABA transaminase, the enzyme responsible for the catabolism of GABA [55]. In addition to enhancing the inhibitory transmission of GABA, other drugs exert their antiepileptic action, also exploiting the blockage of the Ca^2+^ and Na^+^ channels. Among the AEDs that perform their action through these effects are included, felbamate, lamotrigine and topiramate [56]. Other drugs are valproic acid and levetiracetam that perform their mechanism of action enhancing the transmission of GABA and blocking Ca^2+^ channels [57,58].

The anticonvulsant and neuroprotective efficacy of some drugs is also given by the inhibitory action of neurotransmitters, such as glutamate. Glutamate (or glutamic acid) is the most common excitatory neurotransmitter and is responsible for excitatory transmission on neurons. Felbamate and topiramate also perform their mechanism of action inhibiting glutamate thus decreasing l’ hyperexcitability neuronal [59,60].

The choice of drugs is mainly linked to the identification of the type of seizure and epileptic syndrome. For patients with epilepsy, effective seizure control is the determining factor for a good quality of life. AED dosages must be individualized to maximize therapeutic effects and avoid side effects. The early childhood epilepsy syndrome such as DS, LGS and WS present no easy medical management due to the fact that subjects often show convulsion resistant to the available treatment. Therefore, of safe and effective therapies arenecessary to reduce the risk of neurological sequelae. The drugs preferentially used in particular forms of pediatric epilepsy are phenobarbital, phenytoin, benzodiazepine, topiramate, levetiracetam and valproic acid [61].

## 4. Cannabidiol and Molecular Targets in Epilepsy

CBD shows a low affinity for endocannabinoid receptors and it carries out its mechanisms of action by interacting with other molecular targets. One of the most important ion channel targets towards which the CBD shows a high affinity is the Transient Receptor Potential Vanilloid (TRPV). 

Specifically, TRPV1 is a non-selective channel that shows a high Ca^2+^ permeability and is involved in the modulation of seizures and in epilepsy. In fact, when active, it promotes the release of glutamate and the increase in Ca^2+^, with consequent neuronal excitability [62]. The antiepileptic action of CBD does not seem to be due to direct interaction with these molecular targets. However, it has been observed that the CBD agonist action towards TPRV1 determines one a desensitization of these channels with consequent normalization of intracellular Ca^2+^ [63]. T-Type Ca^2+^, are another class of ion channels with which CBD interacts. These channels control Ca^2+^ peaks in neurons and they are involved in the regulation of cell excitability. The activation of these channels due to a hyperpolarization of the membranes of neurons determines an increase in the concentration of intracellular Ca^2+^, in this way the T-Type Ca^2+^ channels increase the excitability of neurons. This mechanism is often observed in pathophysiological conditions such as epilepsy [49]. The interaction of the CBD with the T-type Ca^2+^ channels causes a blockage of these channels, this mechanism could be responsible for the antiepileptic action, even if there are no studies available that confirm this. Receptors represent other molecular targets that have been evaluated to describe their potential role in epilepsy through interaction with CBD. Serotonin receptor (5-hydroxytryptamine [5-HT]) belonging to the superfamily of the G protein-coupled receptors are divided into seven distinct classes (5-HT_1_ to 5-HT_7_). These receptors may depolarize or hyperpolarize neurons, modifying the conductance and/or concentration ionic within the cells. This suggests that 5-HT receptors are involved in epilepsy even though their role is still not entirely clear [64]. CBD shows a high affinity towards two subtypes of serotonin receptors: 5-HT_1A_ e 5-HT_2A_. These receptors can have different functions and regulatory characteristics, in fact, for example, the activation of 5HT_1_ receptors in the hippocampus causes an increase of neurotransmission; in contrast, in raphe nuclei, activation of 5-HT_1A_ receptors produces the inhibition of serotonergic neurons [65]. The dysregulation of brain neurotransmission mediated by 5-HT_2_ might results responsible for the pathophysiology of depression and epilepsy [66]. However, although the role of serotonin receptors in epilepsy is unclear, 5-HT_1A_ e 5-HT_2A_ subtypes may represent a valid therapeutic target through which CBD can perform its anti-epileptic action [61,67]. Opioid receptors (OR) are G-protein-coupled receptors involved in a variety of brain disorders, including epilepsy [68,69]. The CBD at high micromolar concentrations determines the blocking of µ and δ OR, and this block would seem to generate anticonvulsant actions, even if there are still no studies to support this theory. The CBD also shows a good affinity towards the orphan G-protein-coupled receptor (GPR55), a class of receptors involved in the modulation of the synaptic transmission. The agonist action of CBD towards these receptors would seem to attenuate synaptic transmission with consequent antiepileptic effects [70]. 

An important enzyme target of CBD involved in epilepsy is cytochrome P450 (CYP450). CBD inhibits CYP450 [71], but this mechanism does not seem to be directly involved in the antiepileptic mechanism. It seems to be responsible for the hepatic metabolism of a variety of AEDs, as shown by the combined administration of CBD and clobazam (CLB) [72].

## 5. Cannabidiol: Clinical Trials for Epilepsy

In the last decades, some clinical studies were conducted in order to investigate the potential effects of the efficacy of CBD in the management of epilepsy. This review provides an overview of the studies recorded on http://clinicaltrial.gov, distributing them into complete trials (see Table 1) and ongoing trials. All trials test the use of CBD as an adjunct to common AEDs and most of them assess the efficacy and safety of CBD especially in infants, children and teenagers. Trials exploring the combination of CBD/Δ^9^-THC have been excluded. In addition, we included further studies that have been approved by local ethics committees (see Table 2). In the description of these clinical trials, attention was also paid to the possible interactions of CBD with other anti-epileptic drugs.

### 5.1. Completed Clinical Trials

All clinical studies (phases 1, 2 and 3) reported below assess the safety and/or efficacy of CBD in addition to common AEDs. Most of these studies enrolled pediatric patients (0.5 to 17 years) with diagnoses of genetically based epilepsy, LGS, DS and WS, resistant to common antiepileptic treatments. The following trials collect data on administration short-term CBD (from 10 days to 3 months).

A phase 1/2 clinical trial NCT02324673 has evaluated efficacy and safety of the CBD oral solution at three different doses (10, 20 and 40 mg/kg/day) administered for 10 consecutive days in sixty-one children (1–17 years) with drug-resistant forms of epilepsy. It was to assess the plasma concentration of the CBD and its metabolite (7-hydroxycannabidiol, 7-OH-CBD) in blood samples collected at baseline, at day 1 and at day 10 post-treatment at different time points (1, 2, 4, 8, 12, 24, 48, 72 h). On day one, the plasma concentrations of CBD and its metabolite increase in a dose-dependent manner. These levels, instead, decrease at the 10th day. Moreover, both at the first and at the tenth day CBD concentrations are double those of its metabolite. A negative change in clinical global impression of severity score at the end of treatment has shown an improvement of illness in all dosage. An improvement, in a dose-dependent manner, has also been observed in daily seizure activity. Serious Adverse Events (SAEs) such as apnoea, skin eruption and thrombophlebitis, were observed in 5% of patients that received medium-dose CBD and in 9.5% that received high-dose. Further no-serious Adverse Events (AEs), such as anaemia, gastrointestinal disorders (diarrhoea, flatulence, constipation, gastroesophageal reflux disease, nausea), nervous system disorders (somnolence, psychomotor hyperactivity, seizure, ataxia) were respectively observed in 65% of patients treated with low-dose CBD, in 45% treated with medium dose and in 80.95% treated with high-dose. The results of this study showed that CBD can be considered safe and tolerable even at high concentrations.

A phase 2, multi-center clinical trial (NCT02551731) enrolled 9 children (6 months to 36 months) with a diagnosis of WS (Infantile Spasms) who have not responded to first-line therapies. CBD in oral solution was administered at the initial dose of 20 mg/kg/day or 40 mg/kg/day. The protocol provided the division of the study into two parts: Part A and Part B. In Part A was evaluated the efficacy, defined as complete resolution of spasms and hypsarrythmia (if present at baseline) confirmed by video-electroencephalogram (EEG) and safety at day 14 of treatment. Instead, in Part B was evaluated the efficacy and the long-term safety up to 64 weeks of treatment. In Part A, 14.3% of children showed complete resolution of spasms and hypsarrhythmia (when present at baseline) on day 14. Only in 33% showed AEs, while no SAEs were recorded. No results of Part B of the clinical trial were recorded because only one subject has completed the study. Then, these results confirm the efficacy and safety of the CBD oral solution after 14 days of treatment.

The patients that concluded the clinical trials NCT02324673 and NCT02551731 have been involved in the completed phase 3 open-label clinical-trial NCT02318602. The participants were divided for an age range into three groups: infants (1 to <2 years of age), children (2 to <12 years of age) and teenagers (12 to <17 years of age). All individuals continued the treatment with CBD at the same dose of trials NCT02324673 (10, 20 and 40 mg/kg daily) and NCT02551731 (20 mg/kg daily) for 48 weeks. The first outcome of this clinical trial was to evaluate the safety of CBD as adjunctive therapy for children with treatment-resistant convulsive disorders. Patients following treatment with established AEDs were continued uninterrupted, dose adjustments were allowed if necessary based on safety concerns or changes in seizure control. SAEs such as seizures, status epilepticus and mental status changes occurred in 77.78% of infants, in 38.46% of children and 0% of teenagers. No serious AEs (anemia, diarrhoea, constipation, vomiting, infection of the upper respiratory tract, nasopharyngitis, otitis media and influenza) occurred in 88.89% of infants, in 92.31% of children and 88.24% of teenagers. In all patients, no significant changes were observed as respect to baseline in laboratory values or in vital signs. These results show that while the administration of CBD cannot be considered safe in infants, but it was generally well tolerated in adults.

The multicenter, open-label clinical trial, NCT03196934, is an extension of the NCT02318602 trial. The aim of this study is to assess the long-term safety of CBD oral solution as an adjunctive treatment for pediatric subjects with a treatment-resistant seizure. No result is available today.

Six randomized, double-blind, placebo-controlled studies were funded by GW Pharmaceuticals for evaluated the activity of the new formulation of purified CBD oral solution (GWP42003-P or Epidiolex), an epileptic medication and now Food and Drug Administration (FDA) approved for the treatment of seizures associated with DS and LGS in patients two years of age or older.

The first clinical trial GWPCARE1 was divided into two parts: Part A (NCT02091206) and Part B (NCT02091375). NCT02091206, a double-blind randomization study (phase 2), to evaluate the safety of multiple doses of the CBD oral solution (GWP42003-P) in 34 children (4 to 10 years) with DS. All patients before enrolment had to have stabilized all AEDs at least 1 month before and the therapy stability had to be maintained during the study. Participants were randomized to one of the three doses (5, 10 and 20 mg/kg/day) of active drug or placebo at a 4:1 ratio. In addition, patients had to take their usual dose of antiepileptic drugs 2 h before CBD administration. The primary outcome was to assess the incidence of Treatment-Emergent AEs (TEAEs). A pharmacokinetic evaluation was also performed by measuring the plasma concentrations of CBD and its metabolites 7-OH-CBD and 7-carboxycannabidiol (7-COOH-CBD), and of the most common antiepileptic drugs taken by patients. Serious TEAEs, such as pyrexia and convulsions, occurred in five patients: one at the dose of 5 mg/kg/day, two at the dose of 10 mg/kg/day, one at the dose of 20 mg/kg/day and one in the placebo group. No serious TEAEs (such as pyrexia, somnolence, decreased appetite, sedation, vomiting, ataxia and abnormal behaviour) reported in 80% of patients at 5 mg/kg/day of CBD, in 62.5% at the 10 mg/kg/day dose, in 77.78% at the 20 mg/kg/day dose and in 85.71% of placebo group. SAEs such as status epilepticus, convulsion, parvovirus infection, rash maculopapular, occurred in 10% of patients who received 5 mg/kg daily of CBD, in 25% at the 10 mg/kg daily, in 11.11% at the 20 mg/kg daily and in the 14.29% in placebo group. CBD was generally well-tolerated at the 5–20 mg/kg/day dose range. Elevated transaminases (ALT or AST) were only reported with concomitant use of valproate. The study showed that exposure to CBD and its metabolites increased in a dose-dependent manner, and 7-COOH-CBD was the most abundant circulating metabolite at all doses and times. In fact, at the end of treatment, 7-COOH-CBD levels were 13–17 times higher than those of CBD. The results also showed a pharmacokinetic interaction of CBD with CLB, resulting in an increment of the metabolite N-desmethylclobazam [N-CLB] in plasma exposure of the patients. An elevation in N-CLB, was absent in patients co-administered with stiripentol, possibly reflecting prior inhibition of the CYP2C19 isoenzyme [73]. 

All doses of CBD were well-tolerated and the 20 mg/kg/day dose was chosen by the for Part B (NCT02091375) study. NCT02091375 enrolled 120 children (2 to 10 years) with DS and drug-resistant epileptic seizures. Patients received either the CBD oral solution at a dose of 20 mg/kg/day (*n* = 61) or placebo (*n* = 59), for 14 weeks, in addition to the standard antiepileptic treatment. During CBD-treatment, SAEs (status epilepticus, convulsion and somnolence) occurred in 16.39% of patients and in 5.8% of the placebo group. Instead, non-serious AEs (diarrhoea, vomiting, pyrexia, fatigue, upper respiratory tract infection, nasopharyngitis, decreased appetite, somnolence, lethargy, headache, convulsion, cough, irritability, gamma-glutamyltransferase increased, transaminases increased, weight decreased) occurred in 75.41% of patients who had taken CBD at the dose of 20 mg/kg/day and in 47.46% of the placebo group. The results suggested that, following the administration of CBD, the median frequency of seizures decreased from 12.4 to 5.9, compared to a decrease from 14.9 to 14.1 in the placebo-treated group. In 43% of patients treated with CBD and in 27% of patients in the placebo group occurred a reduction in seizure frequency by 50% or more and 3 patients were free of seizures [74]. Although the administration of CBD has caused high rates of AEs, CBD appears to be efficacy in the treatment of patients with DS. 

Subsequently, GW launched a second Phase 3 trial, GWCARE2 (NCT02224703), to evaluate DS patients’ responses to either a low (10 mg/kg/day) or a high dose (20 mg/kg/day) of GWP42003-P for 14 weeks. The study, still recruiting, plans to enroll 150 participants, both children and adults (2 to 18 years). The results are not available. 

The phase 3 clinical trial NCT02224560 (GWPCARE3) included 225 patients with LGS (2 to 55 years) with two or more seizures/week. This study was to evaluate the safety and efficacy of the CBD oral solution (GWP42003-P) as an adjunctive treatment of other antiepileptic drugs. The patients were divided into 3 groups and treated with CBD at the dose of 10 mg/kg/day or 20 mg/kg/day or with placebo for 14 weeks. During the treatment, SAEs such as pneumonia, status epilepticus, elevated aspartate aminotransferase concentration, elevated alanine aminotransferase concentration and elevated γ-glutamyltransferase concentration, occurred in 19.40% of patients treated with 10 mg/kg/day of CBD, in 15.85% treated with 20 mg/kg/day and in 10.53% of the placebo group. Increases in serum aminotransferase concentrations occurred only in patients treated with placebo. While, non-serious AEs (diarrhea, vomiting, decreased appetite, pyrexia, fatigue, somnolence, upper respiratory tract infection, nasopharyngitis) have been observed in 53.73% of 10 mg/kg daily group, in 76.83% of 20 mg/kg/day group, and in 52.63% of the placebo group. A median percent reduction from baseline in drop-seizure frequency was 37.2% in the 10 mg/kg/day CBD group, 41.9% in the 20 mg/kg/day CBD group, and 17.2% in the placebo group. The results reported by the authors show that the addition of the CBD to conventional antiepileptic therapy reduces the frequency of seizures in a dose-dependent manner [75]. 

The phase 3 clinical trial NCT02224690 (GWPCARE4) included 171 patients (aged between 2 and 55 years) with a diagnosis of LGS. Participants had to have taken one or more antiepileptic drugs (the most used was lamotrigine, valproate and CLB) at a stable dose for at least 4 weeks prior to screening as well as interventions for epilepsy. The first endpoint was to aim the efficacy of the CBD oral solution (GWP42003-P) as adjunctive treatment in reducing the number of drop seizures when compared to the placebo. The secondary endpoint was to assess the safety of CBD by measuring AEs using standard severity measures. Individuals were divided into two groups: 85 received placebo and 86 received a CBD at a dose of 20 mg/kg/day for 14 weeks. SAEs (pneumonia, viral infection, alanine aminotransferase increased, aspartate aminotransferase increased, γ-glutamyltransferase increased) occurred in the 23.26% of CBD group and in 4.71% of patients in the placebo group. Serious TEAEs (increased levels of alanine aminotransferase, aspartate aminotransferase and γ-glutamyltransferase) occurred in four patients in the CBD group. Instead, the most common no serious-AEs (vomiting, diarrhoea, loss of appetite and drowsiness) occurred in the 61.63% of CBD group and in 50.59% of patients in the placebo group. After 14 weeks of treatment, the monthly frequency of seizures decreased by a median of 43·9% from baseline in the CBD group. A reduction in seizures frequency of 50% or more, was reported in 44% of patients in the CBD group and in 24% of patients in the placebo group. The study found that in many patients treated with antiepileptic drugs that included CLB, a higher onset of somnolence was observed. High levels of transaminases were recorded in patients treated with valproate. Nevertheless, the high rate of AEs, the results showed that the administration of long-term CBD oral solution in patients with LGS determines the reduction in seizure frequency compared to placebo [76].

Subsequently, all patients who completed the treatment period in NCT02091206, NCT02091375, NCT02224703, NCT02224560 or NCT02224690 were included in the sixth clinical trial GWPCARE5 (NCT02224573). The results of this study will help to understand the safety of CBD administered over long periods. Patients received an oral solution of CBD (100 mg/mL), titrated from 2.5 to 20 mg/kg/day over a 2-week period, in addiction with their existing treatment. The median treatment duration was 274 days. SAEs such as status epilepticus and convulsion occurred in 29.2% of patients. Commonly reported AEs (diarrhoea, pyrexia, decreased appetite and somnolence) occurred in 93.2% of patients. 17.2% of patients from GWPCARE1 that taking valproic acid, had liver transaminase elevations. In patients from GWPCARE1 Part B, the monthly frequency of seizures from baseline decreased by a median of ranged from 38% to 44% in 12-week periods up to week 48.85% of patients reported an improvement in the overall condition after 48 weeks of treatment. This trial showed that long-term CBD treatment was safe and efficacy to reduce seizure frequency in patients with treatment-resistant DS [77].

A randomized controlled trial NCT02565108 (phase 2) included twenty patients (aged 18 to 65 years) with diagnosed epilepsy treated with CLB. This study examined the possible drug-drug interactions between CLB and CBD. Participants before enrolment followed a stable therapy for at least a month with antiepileptic drugs, including CLB. Patients received CBD oral solution (GWP42003-P) at a dose of 20 mg/kg/day after taking CLB for 21 consecutive days. 75% of patients in the CBD group and 50% of patients in the placebo group showed non-serious AEs (diarrhea, nausea, vomiting, dizziness, somnolence, sedation, dermatitis). Results showed that all participants reduced the maintenance dose of CBD of 10%/day. 

Eighteen participants who completed trial NCT02565108 were transferred to the open-label extension (OLE) trial NCT02564952. The OLE phase was a safety study. Initially, all participants received CBD at a dose of 20 mg/kg/day, thereafter the dose was decreased or increased to a maximum of 30 mg/kg/day. All individuals during the study continued to receive CLB. In addition to CLB, participants could not take more than two other AEDs during the study. Only seven of the 18 participants completed the study, 11.11% showed SAEs (status epilepticus, seizure, alanine aminotransferase abnormal, aspartate aminotransferase abnormal γ-glutamyltransferase abnormal). While, 94.44% of patients presented no-serious AEs such as diarrhoea, vomiting, headache, hyponatraemia, dizziness, seizure, somnolence, irritability, respiratory tract infection. The high rate of AEs in the concomitant use of the CBD and CLB for prolonged periods of time may be unsafe.

The clinical trials of phase I NCT02695537 and NCT02700412, will evaluate prospectively and longitudinally the safety and tolerability of CBD oral solution (Epidiolex) at various doses, between 5 mg/kg/day and 25 mg/kg/day with additional titration in some cases up to 50 mg/kg/day. These two trials will enroll both 100 patients with drug-resistant epilepsy. Clinical trial NCT02695537 will enroll patients aged 1 to 18 years, while the NCT02700412 patients aged 17 to 99 years. However, Gaston, et al. [78] evaluated possible CBD interactions with antiepileptic drugs typically used in 39 adults and 42 children of these trials. An analysis was carried out to check for non-uniform changes in both the CBD dose and the dose of other AEDs. In the two combined arms (pediatric and adult) the results recorded linear increases in serum levels of topiramate, rufinamide and [N-CLB] and linear decreases in CLB levels correlate with increasing CBD does. However, there were no significant changes in the levels of other AEDs analyzed (valproate, levetiracetam, phenobarbital, clonazepam, phenytoin, carbamazepine, lamotrigine, oxcarbazepine, ethosuximide, vigabatrin, ezogabine, pregabalin, perampanel and lacosamide). During the study, six adults and eight children showed sedation. The intake of concomitant CBD and valproate resulted in high levels of AST and ALT. Liver function tests showed elevated damage greater than three times the normal limit in four children who dropped out of the study, while the damages of about twice the upper normal limit in eight adults were resolved with valproate withdrawal. A major onset of somnolence following the concomitant administration of CBD and CLB and high levels of transaminases following co-administration of CBD and valproate was also recorded in another study [73] and in clinical trial NCT02224690. In conclusion, the results obtained by the researchers show that the use of CBD with other drugs can be considered safe. On the contrary concomitant use of CBD with valproate is not recommended as a significant liver dysfunction has been observed. Probably because CBD enhances the toxic action of valproate. The interaction between CBD and CLB was also highlighted. Since both of these drugs are metabolized of the cytochrome P450 pathway, this interaction can often induce high plasma levels of n-CLB. Therefore, it is important to monitor this drug-drug interaction. However, as the adverse effects occurring, in this case, are not serious, the concomitant use of CBD with CLB can be considered safe and above all effective, especially in pediatric patients with refractory epilepsy. Part of the results of NCT02695537 and NCT02700412 were described by Szaflarski, et al. [79]. The study showed the efficacy and safety of Epidiolex in 72 children and 60 adults. The results obtained show an average reduction of all types of seizures of 63.6% with difference significant between baseline and 12 weeks. The reduction in seizures seems to have remained stable, in fact, there were no significant differences between 12 and 24 weeks and between 24 and 48 weeks. The severity of the seizures assessed by the Chalfont Seizure Severity Scale (CSSS) also showed an improvement from a baseline score of 80.7 to enroll at 39.3 at 12 weeks with CSSS scores stable even between 12 and 24 weeks and between 24 and 48 weeks. The analysis of AE Profile indicates a significant improvement in the presence/severity of adverse events between the baseline and 12 weeks with stable AEDs thereafter without significant differences between 12 and 24 weeks and between 24 and 48 weeks. The results of this study show significant improvements in the profile of adverse events, in the severity of crises and in reducing the frequency of seizures as early as 12 weeks; improvements that have been maintained during the 48 weeks of treatment.

A clinical trial of phase II NCT02987114, is an open-label, single-center trial, that recruited 16 children (aged 2-15 years), with intractable epilepsy. The aim of this trial was to evaluate the safety, tolerability and efficacy of oral administration of PTL101 (formulation of seamless gelatin matrix green beads containing CBD) as adjunctive therapy for pediatric intractable epilepsy. Patients at least 4 weeks before enrolment had to have stabilized the doses of antiepileptic drugs. This clinical trial has included 4 weeks of observation of clinical parameters and 13 weeks of CBD treatment at an initial dose of 25 mg/kg daily up to the maximum dose of 450 mg/kg. Subsequently the patients were monitored for 2 weeks. The results of this study, not yet available.

The results obtained from all the completed studies show that CBD is a safe compound when combined with common AEDs. An aspect of particular interest concerns the association of CBD with valproate and CLB. In particular, some studies have shown that the association between CBD and valproate leads to a reduction in liver function related to an increase in transaminases. This alteration has been shown to be reversible and not to cause permanent liver damage. Pharmacokinetic studies have shown that CBD determines, as associated with CLB, a plasma increase in the metabolites of this benzodiazepine. All trials reporting efficacy data show that CBD is able to reduce the frequency of seizures.

### 5.2. Ongoing Clinical Trials

In the last 4 years, various tests have been started to evaluate the efficacy and safety of CBD as an antiepileptic. In this subsection, we have collected the trials that to date have not yet available results as they are still in the patient recruitment phase.

Two phase 2 clinical trials NCT03355300 and NCT03336242 are expecting to recruit about 30 pediatric patients (3 to 17 years), with treatment-resistant childhood absence seizure. Both studies will include three experimental treatment cohorts (20, 30 and 40 mg/kg/day). NCT03336242 will assess efficacy, safety, tolerability and pharmacokinetics of CBD oral solution after 4 weeks of treatment. This study will include a 4-week screening period and a 5 or 10 day titration period (depending on study cohort), a 4-week treatment period followed by 5-day tapering for doses >20 mg/kg/day and a 4-week follow-up period. Instead, NCT03355300 will evaluate the long-term (up to approximately 54 weeks) safety and tolerability of the CBD oral solution, monitoring the incidence of SAEs and AEs during and after treatment. For both trials, the final data collection and the results are expected by the end of the year 2019.

In the clinical trial NCT03676049, CBD will be administered as an adjunct to all current AEDs in 5–10 patients (aged between 5 and 19) with refractory epilepsy. The CBD oral solution used for treatment, with prior approval from the National Institute on Drug Abuse was prepared at the University of Mississippi, and subsequently received FDA approval for compassionate use. A dosing titration period will start with 100 mg/day, and will be titrated monthly as tolerated based on clinical response, up to 300 mg/day. During the treatment period the patients will be subjected to control visits at the baseline, at the fourth, at the eighth and at the twelfth weeks. During these visits the efficacy of the treatment will be evaluated, observing the laboratory tests, quality of life of the patient, the profile of the side effects and the crisis count. Patients who after 3 months of treatment show stability could continue the use of CBD for another 3 months. 

The clinical trial NCT02461706 will assess the safety and efficacy of CBD when administered as adjunctive therapy in 50 children (2 to 16 years) who have resistant to AEDs. Patients treated with AEDs were to have stabilized doses at least 4 weeks prior to enrolling. The study established the starting dose of 25 mg/kg/day. Maximum dose titration should be achieved in most patients within 5 weeks. The patients will be clinically evaluated at baseline, once a month for three months and once every three months thereafter. In addition, to ensure the safety of the study, all patients who reached the maximum dose (more than 600 mg of daily) of the CBD will be monitored at least once a month until the steady state of the maintenance dose was reached.

A double-blind, randomized, placebo-controlled phase 3 trial (NCT02783092), is intended to evaluate the efficacy of the adjuvant use of CBD oral solution (200 mg/ml dissolved in corn oil), in patients with epilepsy. The estimated 126 patients (2 to 18 years) will be treated with CBD at the initial dose of 5 mg/kg/day, up to a maximum dose of 25 mg/kg/day. The primary outcome is to evaluate whether CBD treatment resulted in a 50% reduction in seizure frequency compared to treatment with antiepileptic drugs after 30 days. The results of this study (estimated final data collection in August 2020) will make it possible to clarify the efficacy of CBD at different doses.

The phase I clinical trial NCT02286986 is a multi-center study that to investigate the pharmacokinetics and dose-ranging tolerability, efficacy and safety of CBD (GWP42003-P), in 25 children and young adults (2 to 25 years) with epilepsy. The study was divided into two parts: Part A and Part B. Part A was used to evaluate the safety and tolerability of more ascending doses of GWP42003-P compared to placebo. The best-tolerated dose in Part A of the study was used to treat patients in Part B for 60 consecutive days. The antiepileptic efficacy of GWP42003-P compared to placebo was evaluated by monitoring the incidence in convulsions, determining the plasma concentration of GWP42003-P and its main metabolite following the escalation of multiple doses of GWP42003-P. Furthermore, was investigated the effect of GWP42003-P on the pharmacokinetics of concomitant and cognitive function, sleep quality and daytime sleepiness were also observed. The trial is still active and the results have not been published, yet. 

Two phases II clinical trials NCT02607904 and NCT02607891 want to verify the possible drug-drug interactions between GWP42003-P and two antiepileptic drugs, stiripentol or valproate in patients with epilepsy. Both trials will enroll patients between 16 to 55 years. In the trials NCT02607904, patients will be treated up to a maximum dose of 30 mg/kg/day for 12 months. Instead, in NCT02607891 trials, the participants will be randomized into a 4: 1 ratio to receive GWP42003-P or corresponding placebo. The hypothesis is that levels of stiripentol or valproate may be altered as a result of using GWP42003-P. During treatment, CBD will be administered at a maximum dose of 20 mg/kg/day for 25 days. Participants had to take stiripentol or valproate and no more than two other AEDs during the blinded period of the study.

Two phases III clinical trials NCT02953548 and NCT02954887 intended to evaluate the efficacy and safety of CBD oral solution (GWP42003-P; GW Pharmaceutical) in infants with WS (Infantile Spasms). These studies weredivided into 3 phases: a pilot safety phase; a randomized central controlled phase and an open-label extension phase. The NCT02953548 will be described in the pilot phase. Two cohorts of five participants will be enrolled sequentially. GWP42003-P will be administered up to a maximum dose of 40 mg/kg/day for the 2-week treatment period. Instead, clinical trial NCT02954887 will be an extension trial that will recruit 202 infants (from 1 month to 24 months), for 1 year of treatment. The results expected from this study will allow observing if the administration of CBD will be effective in infants with WS. 

Two phases III clinical trial NCT02544763 and NCT02544750 (GWPCARE6) will evaluate respectively, in a double-blinded phase and in an open-label extension phase, the efficacy of the CBD oral solution (GWP42003-P) as adjunctive therapy by monitoring the frequency of seizures in patients with TSC. NCT02544763 is expecting to recruit about 210 patients (1 to 65 years). All AEDs or interventions will be stabilized at least 1 month before the screening and the stability of the therapy will be maintained during the study. Patients will be treated with CBD at the dose of 25 or 50 mg/kg/day for 16 weeks. The efficacy of the CBD will be tested by evaluating the change in seizure frequency. Patients that will complete this blinded phase will be included in the NCT02544750 trial. The safety of CBD administration will be measured based on the incidence of AEs. All participants will be dosed up to a maximum of 50 mg/kg/day. From these two trials the results, not yet available, will help to understand if the administration of CBD can lead to a decrease in the crisis in patients with TSC.

A phase 1/2 clinical trial NCT03014440, aim to determine the safety and tolerability of CBD (Epidiolex) in addition to the anti-epileptic treatments in use, in patients aged 1 to 20 years with drug-resistant epilepsy. Antiepileptic therapy followed by patients had to be stable for at least 1 month. To date, there is no information available regarding the treatment, the doses used and the results. 

The NCT02660255 is an observational, open-label, flexible dose study. The aim of this trial is to evaluate the safety and efficacy of Epidiolex, in addition to common AEDs. The study will be recruited subjects aged 1-60 years with treatment-resistant epilepsy. Patients prior to enrolment will be treated with 1–4 AEDs on stable settings from least 1 month. Epidiolex will be administered for 1 year and 9 months. To date, no superior information and results are available, yet.

The clinical trial NCT02397863 is an open-label, multi-center study including patients (1 to 18 years of age) with drug-resistant epilepsy. Patients are treated with CBD (Epidolex), the daily dosage is up to 25 mg/kg/day with optional up-titration to a maximal daily dosage up to 50 m/kg/day until the end of treatment. Treatment was provided for a total of 52 weeks. For this study the results have not been published, yet. 

Clinical trial NCT02332655 (phase 1/2) aims to assess the tolerability and optimal dose of CBD to be used as a treatment in children and young adults with SWS and drug-resistant epilepsy to define the optimal dose of Epidiolex. The study involving the recruitment of the 10 patients (aged 1 months to 45 years) already in treatment with antiepileptic drugs. Patients treated with 1–5 basic antiepileptic drugs had to have reached stable doses for a minimum of 4 weeks prior to enrolment. Treatment will start with 2 mg/kg/day. The dose will be increased by 3 mg/kg/day after seven days and then by 5 mg/kg/day every seven days up to a maximum dose of 25 mg/kg/day given for 48 weeks. From the expected results potential efficacy of CBD in refractory crises in patients with SWS will emerge.

An open-label observational study NCT02556008 will evaluate the efficacy of pure CBD for the treatment of 25 children (1 to 17 years) with severe refractory epilepsy. The pure CBD used during treatment is not approved by the FDA, therefore, investigators conducted this study through the FDA’s expanded access mechanism for compassionate use. CBD will be administered as an adjunct to all current anti-epileptic therapies. Patients had to undergo therapeutic treatment with 1-3 basic antiepileptic drugs at stable doses for a minimum of 4 weeks prior to enrolment. The expected dosage of the study was 2 mg/kg/day for a first week, 3 mg/kg/day for the second week, 5 mg/kg/day for the third week up to a maximum dose of 25 mg/Kg/day. Seizure frequency will be assessed four weeks before the initiation of CBD, the next month, and at least every 3 months thereafter. The results of efficacy of CBD are not yet available. Data from these studies will be available soon, as the final data collection for many studies is expected by the end of 2019.

### 5.3. Clinical Trials Approved by Local Ethics Committees

In this subsection, clinical studies published in indexed journals have been described. Many of these manuscripts report the results obtained on long periods of treatment with CBD and provide important efficacy data. In addition, the common antiepileptic drugs taken by patients in association with CBD are detailed.

The study conducted by Geffrey, et al. [72], approved by Massachusetts General Hospital (MGH) Institutional Review Board (IRB) evaluated the CBD interaction with CLB. The aim of this study was to evaluate possible interactions between CBD and CLB, assessing its efficacy, safety and pharmacokinetics. For this study, 13 patients (4 to 19 years) with refractory epilepsy and treated with CLB were recruited. Patients started taking CBD at a dose of 5 mg/kg/day and treated up by 5 mg/kg/day each week to a dose of 25 mg/kg/day, for 8 weeks. CLB was administered daily at a stable dose of 0.5 mg/kg that was decreased during the study when side effects were observed. The plasma levels of CBD, CLB and [N-CLB] were measured at baseline and at weeks 4 and 8 of treatment. The results of the efficacy study showed a 50% convulsion reduction in nine out of 13 subjects, corresponding to a 70% response rate. In two patients, however, there was an increase in the frequency of seizures during the treatment period, therefore the dose of CLB was reduced. Increases in plasma levels of CBD, CLB and its metabolite were recorded. Already in the fourth week, the mean of CBL levels had been an increase of 60 ± 80%, while the mean in [N-CLB] was an increase of 500 ± 300%. The results of the safety study show that in 77% subjects AEs were reported as somnolence (*n* = 6), ataxia (*n* = 2), irritability (*n* = 2), restless sleep (*n* = 1), urinary retention (*n* = 1), tremor (*n* = 1) and loss of appetite (n = 1). After adjusting the doses of CLB all AEs were resolved. Therefore, the results reported by the authors show an interaction between CBD and CLB, and that CBD influences [N-CLB] levels much more than CLB levels.

The efficacy of the interaction between CBD and CLB was also seen in a study conducted by Porcari, et al. [80]. This study was approved by the Vanderbilt University Institutional Review Board. In this retrospective study, the aim is to define the efficacy of CBD alone or in association with CLB in 209 children (≤ 19 years) with epilepsy. The duration of treatment in patients receiving CBD was 1.1 years, while patients received CBD + CLB for 1.3 years, and for 2.5 years received CLB. The reduction of antiepileptic drugs was seen in 21% of the CBD group, in 26% CBD + CLB and in 18% of CLB group. No-seizures were observed in 14% of patients in the CBD group, in 9% of patients in the CBD + CLB group and in 11% of patients in the CLB group. The results reported by the authors show a reduction of the crises > of 50% in 33% of the CBD group, in 44% of the CBD + CLB group and in 38% of the CLB group. It was also observed that LGS was the most commonly observed syndrome in all cohorts, and in these patients, the response rate was 58% with CBD, 52% with CBD and CLB and 40% with CLB alone. The sedation was the most common AEs reported in 36% of patients in the CLB group, in 7% of patients in the CBD + CLB group and in 0% of CBD group. This retrospective study suggests that CBD is useful in the treatment of refractory epilepsy with benefits that cannot be attributed to the interaction with CLB and increased levels of its active metabolite. 

Morrison, et al. [81] conducted a pharmacokinetic study that evaluated the possible drug-drug interactions between CLB, stiripentol or valproate and CBD (Epidiolex). This study was approved by the Independent Ethics Committee of the Foundation Evaluation of Ethics in Biomedical Research, Assen, The Netherlands. For this open-label phase I study, were enrolled 78 healthy subjects. The primary outcome was to evaluate the interaction of multiple CBD administration as a perpetrator drug with antiepileptic drugs (victim) at steady-state plasma concentrations: CLB (and its active metabolite, *N*-desmethylclobazam); stiripentol and valproate (and its potentially hepatotoxic metabolite, 4-ene-VPA). On the contrary, was also evaluated the interaction of CBD (victim drug) and its metabolites 7-OH-CBD and 7-COOH-CBD at steady-state plasmatic concentrations, with multiple doses of CLB, stiripentol or valproate as perpetrators drugs. CBD was given at 750 mg twice daily, CLB at 10 mg/kg/day, stiripentol at 750 mg and valproate at 750 mg twice a day. The results showed a significant interaction between CBD and CLB. When CLB was used as the victim drug, significant increases in its metabolite [N-CLB] were recorded. These increases are related to an inhibition of the CPY2C19. In addition, the concentrations of the active metabolite 7-OH-CBD increased when was co-administered with CLB. Stiripentol, however, increased by 28% when it is at steady-state plasma concentrations alone, and by 50% following co-administration with CBD. The 50% increase in stiripentol concentration may be caused by an inhibition of CPY2C19 by the CBD. Instead, co-administration of stiripentol with CBD not caused an increase in CBD concentrations, but caused a 29% increase of 7-OH-CBD and 13% of 7-COOH-CBD. The interaction of the CBD and valproate did not affect the pharmacokinetics of the two drugs. Regarding the safety study, six subjects were withdrawn due to adverse events; three when CLB was added to the steady-state CBD and three when the valproate was added to the steady-state CBD. Two subjects reported SAEs when CLB was co-administered to CBD. Moderate AEs were reported in eight subjects; instead mild AEs were reported in most subjects. The results obtained by the authors can be concluded by saying that the co-administration of drugs was moderately tolerated. Furthermore, the drug-drug bidirectional interaction noted when CLB was co-administered with CBD, suggests a dose reduction for CLB when administered with CBD. 

Another study to evaluate the efficacy of the CBD oral solution (GWP42003-P) as a therapy for drug-resistant epilepsy in TSC was conducted by Hess, et al. [82] (approved by Massachusetts General Hospital Institutional Review Board and U.S. Food and Drug Administration). Of the 56 patients enrolled in this study, only 18 patients (aged 2 to 31 years) were evaluated because they were affected by TSC. At the time of enrolment, patients were taking between one and seven anti-epileptic drugs, such as lacosamide (*n* = 14), CLB (*n* = 10), levetiracetam (*n* = 7), lamotrigine (*n* = 5), valproic acid (*n* = 3) and rufinamide (*n* = 3). Treatment started at a dose of 5 mg/kg/day. This dose was increased by 5 mg/kg/day every week up to the initial maximum dose of 50 mg/kg daily, for 12 months. After the third month of treatment, doses of the CBD and concomitant AEDs could be adjusted monthly in almost all patients in order to optimize seizure control. 15 patients achieved the initial maximum dose of 25 mg/kg/day of CBD, while five achieved the highest dose of 50 mg/kg/day of the CBD, and at this dose, none reported CBD-related AEs. Instead, six patients decreased the dose of CBD during the study in order to alleviate AEs and interactions with concurrent AEDs. 66.7% of patients reported AEs and among them, drowsiness, ataxia and diarrhoea. Three months after the treatment, in four patients a reduction in seizure rate greater than 80% was recorded and one patient became seizure-free and he remained free until the twelfth month. The results also show that in patients took CBD and CLB the response rate after 3 months of treatment was 58.3% against 33.3% in patients who did not take CLB. Given the results reported by the authors in this study, the CBD can be considered valid and safe in the treatment of refractory epilepsy in the TSC.

Five patients enrolled in this study were included in another multicentre analysis of CBD expanded-access conducted by Devinsky, et al. [83] (in 11 epilepsy centers in the USA). The aim of this study was to assess safe, tolerated and effective of CBD (Epidiolex) in children and young adults with severe, intractable, treatment-resistant epilepsy (the most common epilepsy syndrome treated were DS and LGS). This study was approved by the institutional review boards at each study site. CBD was used in addition to anti-epileptic treatment. For this trial, 214 patients were enrolled (1 to 30 years); of these 162 patients after the first dose of CBD were monitored for 12 weeks and were included in the safety and tolerability analysis while 137 patients (64%) were included in the efficacy analysis. Patients started the treatment with CBD at the initial dose of 2–5 mg/kg daily up to a maximum dose of 50 mg/kg daily for 12 weeks. In the safety study, SAEs were observed in 30% of patients. Treatment-related serious AEs, such as status epilepticus, diarrhoea, pneumonia and weight loss were recorded in 20 individuals. Instead, in 79% of patients showed no serious AEs, the most common were decreased appetite, fatigue, somnolence, diarrhoea, convulsions, status epilepticus, sedation, lethargy. After 12 weeks of treatment, results showed a median reduction in monthly motor seizures of 36.5%. In the patients with DS (*n* = 32), the treatment led to a median reduction of monthly motor convulsions of 49%, in 16 patients a reduction of 50%. Instead, for patients with LGS (*n* = 30), an average reduction of 36.8% in motor crises was recorded. Findings obtained from this study showed that CBD seems to reduce the frequency of seizures and also shows an appropriate safety profile, even in patients with DS and LGS. 

Another study conducted by Sands, et al. [84] assessed the long-term safety, tolerability and efficacy of CBD to children with refractory epilepsy. This study was approved by the Human Research Ethics Committee of the UCSF Benioff Children’s Hospital. The CBD oral solution (Epidiolex) was administered in addition to other anti-epileptic treatments in 26 patients (aged 1 to 17 years). The doses of concomitant antiepileptic drugs had to be stable during the 4-week of baseline period and had to remain stable during the first three months of treatment. CBD was administered at the starting dose of 5 mg/kg daily and subsequently, weekly dosage was measured in increments of 5 mg/kg daily up to a maximum dose of 25 mg/kg daily. The duration of therapy ranged from 4 to 53 months. The patients underwent blood tests performed during the baseline period, after 1, 2 and 3 months and thereafter every 3 months from treatment. Furthermore, the minimum concentrations of antiepileptic drugs were evaluated. The frequency of seizures and AEs was monitored during the treatment period. The primary outcome of the study was to test the efficacy of CBD in terms of > 50% reduction in the frequency of motor seizures. Fifteen of 26 patients discontinued treatment, one due to a status epilepticus, one for severe weight loss, all others for lack of efficacy. Instead, six patients showed SAEs as status epilepticus (*n* = 3), catatonia (*n* = 2) and hypoalbuminemia (*n* = 1). 21 out of 26 patients reported no serious AEs among which the most frequent were: reduced appetite (*n* = 10), diarrhoea (*n* = 9), and weight loss (*n* = 8). In patients showing significant weight loss, the doses of CBD were reduced. Changes in the concentrations of antiepileptic drugs were observed in four patients. Three of them reported increased CLB concentrations, one reported an increment in phenobarbital contractions. In three patients was observed an increment in aspartate aminotransferase and alanine transferase levels when CBD was co-administered with valproate. The reduction in the frequency of seizures > 50%, was rediscovered in 38.4% of patients after 3 months of treatment, in 56.7% after 6 months, in 42.3% after 9 months, in 38.4% after 12 months, 42.3% after 18 months and 34.6% after 24 months. In conclusion, after 24 months of treatment, of the 26 patients enrolled, only nine continued CBD as adjunctive therapy. Of these patients, seven had a 50% reduction in the frequency of motor crises, three of which remained completely free of seizures. Only seven of the nine patients who continued treatment showed a reduction in seizure frequency > 50% after 36 months. The results reported by the authors showed that long-term CBD results in a clinically significant reduction in seizure frequency, and a low percentage of SAEs. Moreover, because treatment was stopped after a few months in most patients, the number of patients exposed to CBD for a long time is low and the rate of adverse effects over time may be underestimated.

Using the same CBD formulation (Epidiolex) and the same administration doses Kaplan, et al. [85] conducted a study that was approved by the Federal Drug Administration for the use of Epidiolex in the treatment of pediatric medically refractory epilepsy in SWS. In this study, five patients (aged 1 months to 45 years) were enrolled. The maximum dose of 25 mg/kg daily was tolerated only by two patients, while in the other three the maximum tolerated dose was 20 mg/kg per day. Three participants withdrew from the study, two due to lack of efficacy (week 38 and week 9), while one due to the temporary increase in seizures during dose titration, but later re-enrolled. Three subjects remain in the extension phase of the study continued to take CBD for more than a year. All subjects reported at least one CBD-related adverse event during the study such as temporarily increased seizures, behavioral issues, increased aspartate aminotransferase and fatigue. All transient AEs resolved spontaneously after dose changes in concomitant anticonvulsants or CBD. Seizure reduction above 50% was seen in two patients at weeks 14 and in three patients with bilateral brain involvement. Instead, subjects reported improvements in quality of life during the treatment. As suggested by the results obtained from this study, CBD appears to be well tolerated and a valid candidate as adjunctive therapy for seizures management in individuals with SWS. 

Rosenberg, et al. [86] belonging to the same research group of Devinsky, et al. [83] prolonged the study for evaluating the Quality of Life of Childhood Epilepsy (QOLCE) before and after treatment with CBD (Epidolex). The study was approved by the NYU Langone Medical Center institutional ethics board. For this study were enrolled patients (aged 1–30) with intractable treatment-resistant epilepsy. In addition to the baseline antiepileptic drugs, patients were given CBD at the initial dose of 2–5 mg/Kg daily up to the maximum dose of 50 mg/Kg daily for 12 weeks. After 12 weeks of treatment with CBD, the median monthly seizures frequency was 13.9 and the median percent change from baseline was –39.4%. In addition, the results indicated an improvement of 8.2 ± 9.9 points in patient QOLCE. In fact, patients showed an improvement in behaviour, in memory, in energy/fatigue, in control/impotence, in other cognitive functions and in global quality of life.

The same research group of Devinsky, et al. [87], conducted a study for evaluating the safety and efficacy of long-term CBD administration in patients with severe childhood-onset epilepsy, and with CDKL5 deficiency disorder and Aicardi, Doose syndromes and Dup15q syndromes. This study was approved by the IRB at each institution. For this study, 55 patients aged between 1 and 30 were enrolled (with 55 in the safety group and 50 in the efficacy group). Patients were given a pharmaceutical compound of Highly purified CBD (Epidolex). Treatment included 144 weeks of Epidiolex administration in addition to anti-epileptic therapies at the starting dose of 5 mg/Kg per day. During treatment, an increase of 2–10 mg/kg per day was carried out every two weeks up to the maximum dose of 50 mg/kg per day. The efficacy study showed that the percent change in median monthly convulsive seizure frequency for all patients after treatment decreased from baseline of 51.4% to week 12 and of 59.1% to week 48 with a no significant change between weeks 12 and 48. After 12 weeks of follow-up was reported a decrease of 50% or more of seizures in 50% of patients and in 57% at 48 weeks. The safety results of the drug showed that of the 55 patients,10 patients withdrew by week 48, including 5 by weeks 12 and 48 due to lack of efficacy (*n* = 4) and AEs (*n* = 1). A total of 15 (27%) participants withdrew by week 144 of extended follow-up. There were no deaths during the study. SAEs that occurred during treatment were convulsions (9%), status epilepticus (9%) and respiratory infection (5%). While other adverse events reported more frequently were diarrhea (29%), drowsiness (22%) and fatigue (22%). These results can demonstrate the safety and tolerability of long-term treatment with CBD and the reduction in seizure frequency in these four aetiologies of epilepsy.

Chen, et al. [88] conducted an open-label study, the aim was to assess the tolerability and safety of CBD (Epidiolex) in the treatment of drug-resistant epilepsy in children. Sydney Children’s Hospital Network Human Research Ethics Committee approved the protocol for this study. Children (*n* = 40; mean age 8.5 years) with drug-resistant epilepsy and uncountable daily seizures in focal/multifocal epilepsy, epileptic encephalopathy, LGD and DS, were enrolled. CBD was administrated in addition to anti-epileptic therapy at the initial dose of 5 mg/Kg daily for 12 weeks. The initial dose was increased every week by 5 mg/Kg daily up to a maximum dose of 25 mg/Kg daily. During the treatment five children withdrew from the study, two because he had an increase in the frequency of the seizures, one because has manifested significant somnolence, one for respiratory depression and one because their transaminase level was elevated. SAEs occurred in 15 out of patients, the frequent recurring to treatment were increased seizure number (in eight patients), intercurrent illness (in five patients), liver function disorder (in all patients), hyperlipidemia (in all patients), severe somnolence with anorexia and respiratory depression (in one patient). Over-therapeutic phenytoin levels are another SAEs manifested in two participants were considered related to treatment and occurred at doses of 10 mg/kg/day and 20 mg/kg/day. All participants showed AEs not all attributable to treatment. While, AEs that occurred frequently (15 individuals) and linked to the treatment was the drowsiness (AEs spontaneously resolved the 10 participants), and gastrointestinal disorders (nausea, vomiting, diarrhoea) in nine patients, somnolence (13 individuals) and increased seizures (two individuals). Instead, 12 children showed an improvement in health in general. The results reported in this study show that Epidiolex can be considered useful as adjuvant therapy. The presence of adverse events and possible interactions with antiepileptic drugs are important aspects to be taken into consideration.

Szaflarski, et al. [89] conducted an open-label, Expanded-Access Program (EAP) in 25 epilepsy centers in the USA, and it was approved by an institutional review board at each site. The aim of this study was to evaluate the safety and efficacy of CBD oral solution (Epidiolex), in addition to common AEDs in patients with different forms of treatment-resistant epilepsies (TREs). For the study, 607 patients with a mean age of 13 were enrolled. All patients were included in the safety study, while 508 were included in the efficacy study. Treatment involved a 4-week baseline period followed by a 96-week treatment period. During treatment, patients received Epidiolex at the initial dose of 2–10 mg/Kg up to a maximum dose of 25-50 mg/Kg daily. 146 patients (mostly due to lack efficacy [15%] or AEs [5%]) from the safety study group and 136 patients (mostly due to lack efficacy [15%] or AEs [4%]) from the efficacy group were withdrawn from the study. SAEs were found in 33% of patients such as convulsion (9%), status epilepticus (7%), pneumonia (5%), and vomiting (3%). Instead, AEs manifested in 88% of patients the most common were diarrhea (29%), somnolence (22%), and convulsion (17%). Already after 12 weeks of treatment, the median monthly frequency of seizure convulsions was reduced by 51% and by 48 % the frequency of total seizures. These reductions remained stable during the 96 weeks of treatment. Between weeks 12 and 96 the average dose of CBD was 25 mg/kg daily, 55% of patients at follow-up had reduced the dose. Half of the patients taking concomitant CLB and valproate reduced the dose compared to baseline during the study. While, most of those who take simultaneously levetiracetam, had remained at their basal doses. The data obtained from this study show that CBD as an adjunct treatment to common AEDs can be used in the long-term effective treatment in patients with TRE. 

A very interesting data in these studies is the pharmacokinetics and interaction of CBD with common AEDs. The interaction of these drugs is very complex and is linked to the individual metabolites produced and to the possible metabolic pathways that are involved. Specifically, the study conducted by Geffrey, et al. [72] shows a bidirectional drug-drug interaction when CBD is administered with the CLB for long period of time. Therefore, CLB determines an increase in serum levels of the CBD metabolite (7-OH-CBD) and conversely, CBD causes an increase in the metabolite of CLB (n-CLB). CBD is an inhibitor of CYP2C19, an enzyme involved in the degradation of n-CLB, these explain how the CBD associated with CLB causes elevated plasma levels of this metabolite. In contrast, CBD does not influence the pharmacokinetics of valproate and stiripentol when co-administered, moreover, stiripentol causes a slight decrease in CBD metabolites (7-OH-CBD 7-COOH-CBD) while valproate causes a slight increase of 7-OH-CBD. The mechanisms by which these interactions take place are not yet known but do not cause clinically relevant effects. However, the CBD shows good safety profiles, the interaction with these two drugs does not require the interruption of therapy. The modulation of the dose of these AEDs will be sufficient to resolve the adverse events. In conclusion, the results of these studies show that the administration of CBD as an addition to the common AEDs for long periods of time leads to clinically significant reductions in the frequency of convulsive and total seizures in different etiologies of epilepsy. Furthermore, an improvement in the quality of life of these patients was also observed.

## 6. Conclusions

The CBD is a compound extensively studied for its potential efficacy for the treatment of epilepsy. In this review, we reported the studies conducted in infants, children and teenagers affected by epilepsy resistant to common AEDs.

To date, available safety data show that the administration of CBD associated with other AEDs causes non-serious adverse events, which can be resolved reducing the dose of CBD and/or common AEDs. In this context, particular attention should be paid when CBD is associated with valproate and CLB. Specifically, abnormal liver function was noted in participants taking concomitant valproate, therefore, it is necessary to monitor serum levels of these compounds and their respective metabolites. Instead, when CBD is associated with CLB it induces an increase in its metabolites. Since the adverse effects are not serious, this association can be considered safe.

The available results also highlight the efficacy of CBD as adjunctive to common AEDs. The mechanism by which CBD interacts with other AEDs is not yet fully known, as many metabolic pathways involved in this interaction are still unknown. In addition, not all the molecular targets used by the CBD to exercise its antiepileptic action are yet known. However, the results obtained to date encourage the use of CBD associated with AEDs.

## Figures and Tables

**Table 1:** Completed cannabidiol clinical trials in epilepsy (https://clinicaltrials.gov/). The table shows the efficacy and safety of CBD in different forms of epilepsy. In all studies, CBD is used as adjunctive therapy to conventional antiepileptic drugs.

Identifier	Study Title	Subjects	Conditions	CBD Dose	Concomitant AEDs	Efficacy	Security	Ref
NCT02987114	A Study to Evaluate the Safety, Tolerability and Efficacy of Oral Administration of PTL101 (Cannabidiol) as an Adjunctive Treatment for Pediatric Intractable Epilepsy	16 children (2 to 15 years)	Pediatric Intractable Epilepsy	25–450 mg/kg/day	-	-	-	-
NCT02324673	Cannabidiol Oral Solution in Pediatric Participants With Treatment-resistant Seizure Disorders	61 children (1 to 17 years)	Resistant Seizure Disorders	10, 20, 40 mg/kg/day	-	Improvement of illness	SAEs in 5% of patients with medium-dose and in 9.5% with high-dose	-
NCT02551731	Cannabidiol Oral Solution for Treatment of Refractory Infantile Spasms	9 infants (6 to 36 Months)	Refractory Infantile Spasms	20–40 mg/kg/day	-	Complete resolution of spasm in 14.3% of children after 14 days of treatment	No SAEs were recorded	-
NCT02318602	Cannabidiol Oral Solution as an Adjunctive Treatment for Treatment-resistant Seizure Disorder	52 children and young adults (1 to 18 years)	Treatment-resistant Seizure Disorder	10, 20, 40 mg/kg/day	-	-	SAEs in 77.78% of infants, in 38.46% of children and in 0% of adults	-
NCT02224703	GWPCARE2 A Study to Investigate the Efficacy and Safety of Cannabidiol (GWP42003-P) in Children and Young Adults With Dravet Syndrome	150 children and young adults (2 to 18 years)	Dravet Syndrome	10, 20 mg/kg/day	-	-	-	-
NCT02695537	Safety, and Tolerability of Epidiolex In Patients (Ages 1–19 Years) With Intractable Epilepsy	100 children and young adults (1 to 18 years)	Intractable Epilepsy	5–50 mg/kg/day	CLB, Valproate, Levetiracetam, Phenobarbital, Clonazepam, Phenytoin, Carbamazepine, Lamotrigine, Oxcarbazepine, Ethosuximide, Topiramate, Vigabatrin, Zonisamide, Eslicarbazepine, Ezogabine, Pregabalin, Perampanel, Rufinamide, Lacosamide	-	4 children with concomitant valproate showed elevate damage of liver function	[78]
						Reduction of seizures of 63.6% after 12 weeks of treatment	Improvement of AE Profile	[79]
NCT02700412	University of Alabama at Birmingham (UAB) Adult CBD Program	100 children and adults (15 to 99 years)	EpilepsySeizures	5–50 mg/kg/day		-	4 children with concomitant valproate showed elevate damage of liver function	[78]
						Reduction of seizures of 63.6% after 12 weeks of treatment	Improvement of AE Profile	[79]
NCT02224560	Efficacy and Safety of GWP42003-P for Seizures Associated With Lennox-Gastaut Syndrome in Children and Adults (GWPCARE3)	225 children and adults (2 to 55 years)	Epilepsy Lennox Gastaut Syndrome	10, 20 mg/kg/day	CLB Valproate Lamotringine Levetiracetam Rufinamide	The median percent reduction in seizures frequency from baseline was 37.2% in the 10 mg/kg/day CBD group; 41.9% in the 20 mg/kg/day CBD group	SAEs were reported in 19.40% of patients at the dose of 10 mg/kg/day of the CBD and in 15.85% at the 20 mg/kg/day	[75]
NCT02091206	A Dose-ranging Pharmacokinetics and Safety Study of GWP42003-P in Children With Dravet Syndrome (GWPCARE1)	34 children (4 to 10 years)	Dravet Syndrome	5, 10, 20 mg/kg/day	CLB Valproate Stiripentol Levetiracetam Topiramate	-	TEAEs in 5 patients; SAE in 10% of patients at the dose of 5 mg/kg/day, in 25% at the 10 mg/kg/day and in 11.11% at the 20 mg/kg/day dose. 6 patients with concomitant valproate had elevated ALT or AST	[73]
NCT02091375	Antiepileptic Efficacy Study of GWP42003-P in Children and Young Adults With Dravet Syndrome (GWPCARE1)	120 children, young adults (2 to 18 years)	Dravet Syndrome	20 mg/kg/day	CLB Valproate Stiripentol Levetiracetam Topiramate	The median frequency of seizures decreased from 12.4 to 5.9, compared to the placebo-treated group	SAEs in 16.39% of patients	[74]
NCT02224690	A Study to Investigate the Efficacy and Safety of Cannabidiol (GWP42003-P; CBD) as Adjunctive Treatment for Seizures Associated With Lennox-Gastaut Syndrome in Children and Adults (GWPCARE4)	171 children and adults (2 to 55 years)	Lennox-Gastaut Syndrome	20 mg/kg/day	CLB Valproate Lamotrigine Levetiracetam Rufinamide	The monthly frequency of seizures decreased by a median of 43,·9% from baseline in the CBD group	Serious TEAEs occurred in 4 patients;SAEs in 23.26% of patients. 16 of the 36 patients on valproate had transaminase elevations	[76]
NCT02224573	GWPCARE5 - An Open Label Extension Study of Cannabidiol (GWP42003-P) in Children and Young Adults With Dravet or Lennox-Gastaut Syndromes	264 children, and adults (2 years and older)	Dravet Syndrome Lennox-Gastaut Syndrome	-	CLB Valproate Stiripentol Levetiracetam Topiramate	The monthly frequency of seizures decreased by a median ranged from 38% to 44%	SAEs in 29.2% of patients	[77]
NCT02565108	A Randomized Controlled Trial to Investigate Possible Drug-drug Interactions Between Clobazam and Cannabidiol	20 adults (18 to 65 years)	Epilepsy	20 mg/kg/day	CLB	All participants reduced the maintenance dose of CBD from 10% for the day	2 patients withdrew from the study due to SAEs (seizure cluster)	-
NCT02564952	An Open-label Extension Study to Investigate Possible Drug-drug Interactions Between Clobazam and Cannabidiol	18 adults (18 to 65 years)	Epilepsy	Initial 20 mg/kg/d titrated to maximum dose of 30 mg/kg/day	CLB	-	SAEs in 11% of patients	-

CBD: Cannabidiol; TEAEs: Treatment-emergent adverse events; SAEs: serious adverse events; AST: aspartate transferase; ALT: alanine transferase.

**Table 2:** Data obtained from trials authorized by local ethics committees (https://www.ncbi.nlm.nih.gov/pubmed/). The table shows the efficacy and safety of CBD in different forms of epilepsy. In all studies, CBD is used as adjunctive therapy to conventional antiepileptic drugs.

Study Design	Subjects	Conditions	CBD Dose	Concomitant AEDs	Efficacy	Safety	Ref
A prospective, open-label, expanded access study	214 children and adults (1 to 30 years)	Drug Resistant Epilepsy	Initial 2–5 mg/kg/day titrated to maximum dose of 50 mg/kg/day	CLB Valproate	The median reduction in monthly motor seizure was of 36.5%	Treatment-related SAEs were recorded in 20 patients; SAEs were reported in 30% of patients. Thrombocytopenia and elevated liver function test in patients with concomitant valproate	[83]
A prospective, open-label study	Children and adults (1 to 30 years)	Drug Resistant Epilepsy	Initial 2–5 mg/kg/day, titrated to maximum dose of 50 mg/kg/day	-	Overall quality of life significantly improved in 48 patients, The median monthly seizures frequency was 13.9	-	[86]
A prospective, multicentre, open-label study	55 children and adults (1 to 30 years)	Epilepsy Dravet Syndrome CDKL5 deficiency disorder Aicardi Doose syndromes Dup15q syndromes	Initial 5 mg/kg/day titrated to maximum dose of 50 mg/kg/day	CLB Valproic acid Levetiracetam Rufinamide Felbamate Topiramate	Median monthly convulsive seizure frequency decreased from baseline by 51.4% at week 12 and by 59.1% at week 48	A serious treatment-emergent AEs such as status epilepticus (9%) and respiratory infection (5%)	[87]
A prospective, open-label study	40 children (1 to 17 years)	Drug Resistant Epilepsy	Initial 5 mg/kg/day titrated to maximum dose of 25 mg/kg/day	-	12 patients reported substantial improvement of the condition	4 patients withdrew from the study because of AEs; SAEs were reported in 15 patients	[88]
A prospective, multiple center, open-label study	607 children (average age 13 years)	Drug Resistant Epilepsy	Initial 2–10 mg/kg/day to maximum dose of 50 mg/kg/day		A median monthly seizure frequency of 51% was recorded after 12 months of treatment and maintained at weeks 96	SAEs were reported in 33% of patients;	[89]
Expanded access program	5 infants (1 to 45 months)	Sturge-Weber Syndrome	2–25 mg/kg/day	Levetiracetam Valproic acid Felbamate CLB Rufinamide Perampanel Clorazepate Oxcarbazepine Lacosamide Topiramate	50% of seizures reductions in all patients; Improvements in quality of life in all patients	AEs were recorded during the study	[85]
Retrospective study	210 children (≤ 19 years)	Epilepsy	2.9, 5.8 mg/kg/day	CLB	50% in seizures reduction in 33% of patients in the CBD group; in 44% of CBD + CLB and in 38% of CLB group	AEs in 36% of patients in the CLB group and in 7% of patients in CBD + CLB group	[80]
Expand access investigational new drug (IND) trial	13 children and young (4 to 19 years)	Refractory Epilepsy	5–25 mg/kg/day	CLB	50% of reduction in seizures in 69.23% of patients	No serious AEs in 77% of patients	[72]
Open-label, fixed-sequence trial	78 healthy subjects	-	750 mg twice daily	CLB Stiripentol Valproate	-	Moderate AEs in 8 patients; mild AEs in most of patients	[81]
Expanded access study	18 children and adults (2 to 31 years)	Tuberous Sclerosis Complex	5–50 mg/kg/day	CLB Lacosamide Levetiracetam Lamotrigine Valproic acid Rufinamide	4 patients recorded a reduction in seizure rate greater than 80%; 1 patient became seizures-free	AEs in 66.7% of patients	[82]
Expanded access program	26 children (1 to 17 years)	Refractory epilepsy	5–25 mg/kg/day	CLB	A 50% reduction in seizures	SAEs in 23.1% of patients	[84]

CBD: Cannabidiol; TEAEs: Treatment-emergent adverse events; SAEs: serious adverse events.
